# Assessing Knowledge, Perception, and Management Toward Traumatic Tooth Avulsion Among Dentists in Jeddah, Saudi Arabia: A Cross-Sectional Study

**DOI:** 10.7759/cureus.46337

**Published:** 2023-10-01

**Authors:** Abdullah A Albassam

**Affiliations:** 1 Department of Endodontics, Faculty of Dentistry, King Abdulaziz University, Jeddah, SAU

**Keywords:** dental education, urgent dental care, traumatic dental injuries (tdis), etiology of dental trauma, pediatric dental trauma, tooth avulsion

## Abstract

Introduction: The long-term prognosis of teeth that have experienced traumatic avulsion is highly dependent on appropriate and prompt emergency management taken immediately after the avulsion. The main goal in the treatment of traumatic permanent tooth avulsion is to replant as soon as possible in its correct anatomical position to maintain the viability of the periodontal ligament and neurovascular supply while restoring aesthetic and preserving the functional integrity of the tooth. General dentists can deal properly with emergency cases, such as tooth avulsion, and also should be aware of appropriate management to take care of avulsions and to determine the prognosis especially since such incidents often occur at times when dental professionals are least prepared to address them.

Objective: The primary aim of this study was to evaluate the knowledge of dentists who are working in various hospitals, clinics, and dental schools in Jeddah, Saudi Arabia, with respect to traumatic permanent tooth avulsion and the appropriate methods for its management.

Methods: The study design is a cross-sectional study with dentists working in various clinical settings in Jeddah, Saudi Arabia. The questionnaire used in the study consists of a total of 19 items: nine items about demographic data, including training background, and 10 multiple-choice questions regarding dental trauma and its management.

Conclusion: Higher dental education and post-graduate training gave the dentists better judgment in dealing with dental emergencies compared to general dentists. Thus, the recommendation of this study is to include the comprehensive treatment of avulsion emergencies in the dental school curriculums and increase the exposure of dental students to such cases.

## Introduction

Traumatic tooth avulsion refers to the complete displacement of a tooth from its natural socket within the alveolar bone. It typically occurs as a result of physical trauma or injury, leading to aesthetic, psychological, and economic problems [1]. These types of trauma may result from various factors, such as falls, car accidents, and sports incidents, with falls being the most frequent cause [2,3]. Tooth avulsion is among the most critical dental injuries [1,4]. The long-term prognosis depends significantly on appropriate and prompt emergency management immediately after the avulsion incident [4]. The primary objective when treating a traumatic avulsed permanent tooth is to reposition it promptly and accurately into its original anatomical location. This is done to ensure the continued viability of the periodontal ligament (PDL) and the neurovascular supply while simultaneously restoring the tooth's aesthetic appearance and preserving its functional integrity [5,6], except in cases when replanting is not applicable (e.g., severely decayed or periodontally affected teeth or medically compromised patients) [3].

General dentists can be proficient in dealing with emergency cases, such as tooth avulsion, and should be knowledgeable about the appropriate management, as they are often the first line of defense. Numerous studies conducted in various countries have assessed the level of knowledge among general dentists regarding the management of avulsed teeth. Several published surveys [1,4,7,8,9,10,11,12] have explored general dentists' understanding of this matter. In a study conducted in Riyadh, Saudi Arabia, the results indicated that a notable proportion of general dentists possessed a moderate level of understanding regarding traumatic tooth avulsion and the correct methods for its management. In addition, it was observed that these dentists lacked awareness of the recommended duration of follow-up care following tooth replantation. Among the surveys conducted, very few targeted dentists in Saudi Arabia. The results of that survey showed that participants had moderate knowledge regarding the management of avulsion. Participants were based in Riyadh with the majority being Saudi general dentists. Consequently, considering the scarcity of published studies on dentists' knowledge regarding tooth avulsion and its management in Jeddah, it is suggested that further research be conducted to assess the knowledge of dentists in Jeddah regarding traumatic permanent tooth avulsion and its appropriate management [7]. This data could be valuable for sharing with healthcare authorities in Jeddah, Saudi Arabia, to enhance awareness and improve the quality of dental care provided by dentists in the region.

## Materials and methods

**Figure 1:**
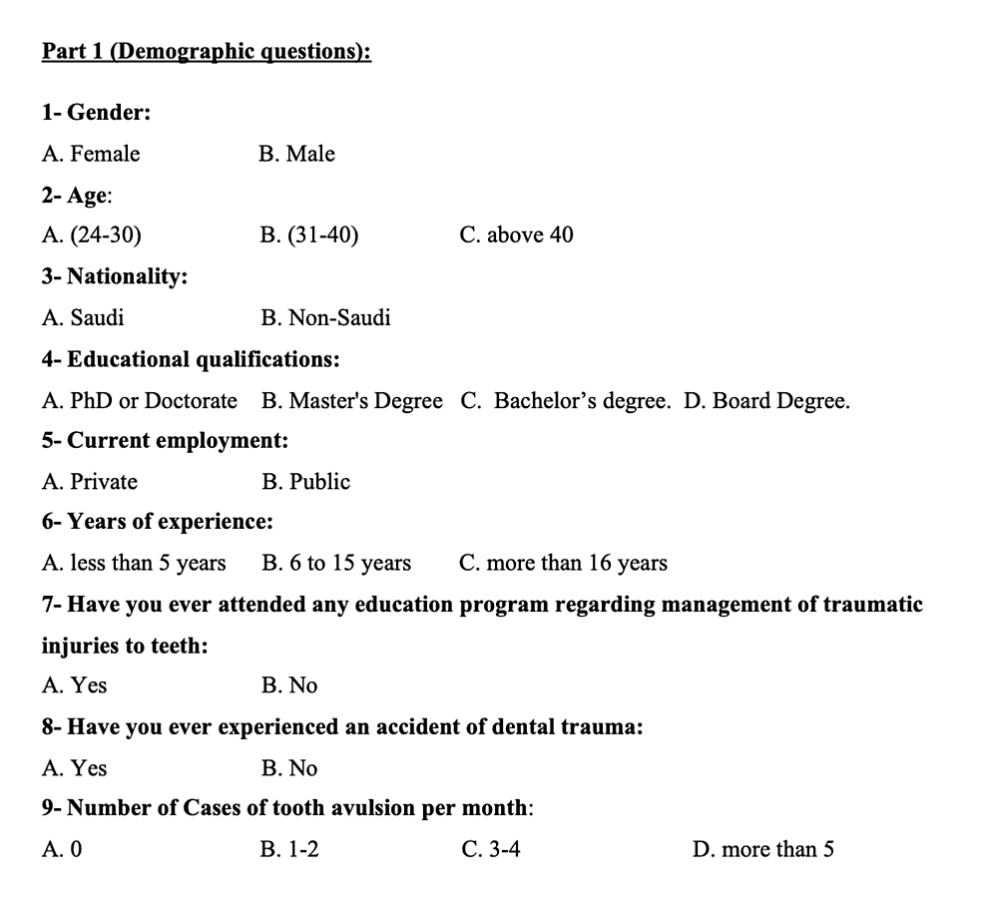
Demographic questions

**Figure 2:**
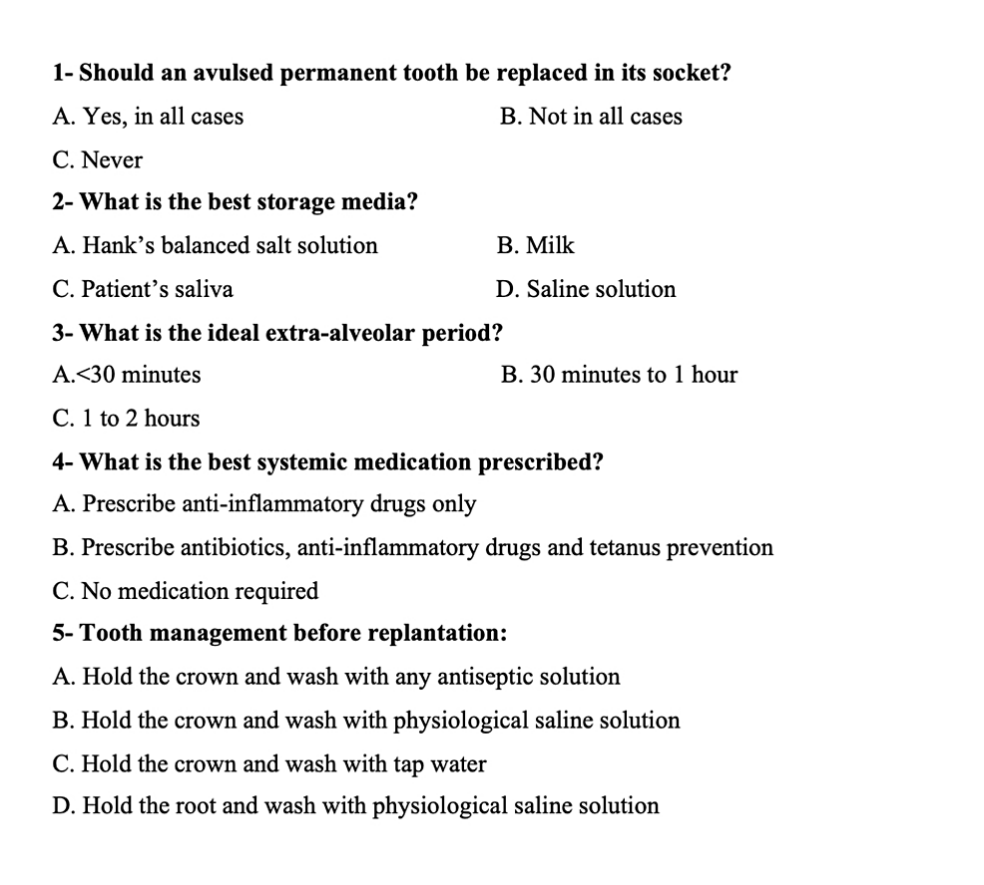
Questions about avulsion and its management (part 1)

**Figure 3:**
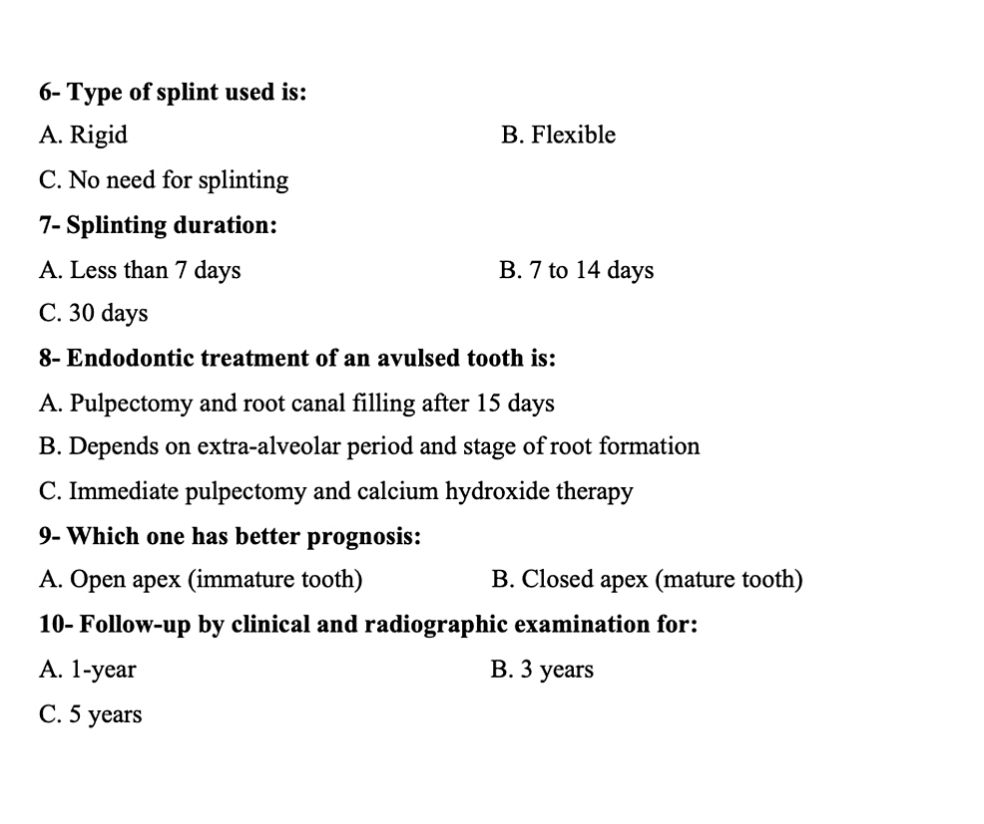
Questions about avulsion and its management (part 2)

This is a cross-sectional study with dentists working in various hospitals, clinics, and dental schools in Jeddah, Saudi Arabia. A 19-item English-language questionnaire was created and pretested. During the pretesting phase, certain challenges were noted in terms of the respondents' understanding of the questionnaire. This questionnaire was adopted from a questionnaire employed by Westphalen et al. in 2007 [12], which underwent development and pretesting within a sample comprising both Saudi and non-Saudi dentists. During the pretesting phase, issues related to respondents' understanding of the questionnaire surfaced. The questionnaire encompassed demographic inquiries and multiple-choice questions concerning dental trauma and its management. Subsequent to identifying these potential challenges, minor adjustments were introduced based on the feedback received. Subsequently, minor adjustments were implemented based on the feedback received from the pretest. The final version of the questionnaire comprised nine demographic items (Figure 1) and 10 multiple-choice questions specifically addressing dental trauma and its management (Figures 2, 3).

The questionnaire was prepared in Google Forms (Google, USA) and the link was emailed to practicing dentists working in various hospitals, clinics, and dental schools in Jeddah, Saudi Arabia. The emails of the participants were provided by the alumni office at King Abdulaziz University database. Dental students, dental interns, dental hygienist, and dental assistants were excluded. Furthermore, handouts were distributed to the attendees of meetings and conferences who were encouraged to participate in the study, if they were dentists practicing in Jeddah.

Qualitative data were expressed in the form of frequencies and percentages. Knowledge scores, on the other hand, exhibited a distribution that deviated from normality, displaying non-parametric characteristics. These data were presented in terms of their median, range, mean, and standard deviation (SD) values. To facilitate comparisons between two groups, the Mann-Whitney U test was employed. Conversely, when the need arose to compare more than two groups, the Kruskal-Wallis test was utilized. In pursuit of a multivariate analysis, a linear regression analysis was conducted to discern significant predictors of knowledge scores. The predetermined significance level was established at P ≤ 0.05. The entire statistical analysis was carried out using IBM SPSS Statistics for Windows, version 23 (released 2015; IBM Corp., Armonk, New York, United States).

## Results

Descriptive statistics

Demographic Data

The present study was conducted with a total of 500 dentists who were contacted to take part in the study. In total, 392 responses were collected (response rate = 78%). There were 208 males (53.1%) and 184 females (46.9%). More than three quarters of the participants were 24-30 years old; approximately one-fifth of the participants aged 31-40 years, while only 3.1% were over 40 years old. The most prevalent degree was bachelor’s degree in more than three-quarters of the participants, followed by master’s, PhD, and board degrees, which showed the lowest prevalence. More than three-quarters of the participants were Saudis. Approximately half of the participants are employed in public hospitals. The majority of the participants (83.9%) had less than five years’ experience, 12.5% had six to 15 years' experience, and only 3.6% had experience of more than 16 years (Table 1).

**Table 1:** Frequencies (n) and percentages (%) of the demographic data of the study participants (n = 392)

Demographic data	n	%
Gender		
Male	208	53.1
Female	184	46.9
Age		
24-30 years	312	79.6
31-40 years	68	17.3
>40 years	12	3.1
Qualifications		
Bachelor	320	81.6
Board	18	4.6
Master	31	7.9
PhD or Doctorate	23	5.9
Nationality		
Saudis	310	79.1
Non-Saudis	82	20.9
Employment		
Public	220	56.1
Private	172	43.9
Experience		
Less than 5 years	329	83.9
6-15 years	49	12.5
>16 years	14	3.6

Education and Practice of Traumatic Injury Management

**Figure 4:**
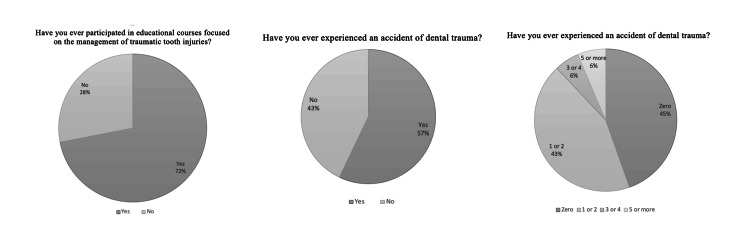
Education and practice of traumatic injury management

Approximately half of the participants in the study had attended an educational course focused on the management of traumatic injuries to the teeth. More than half of the participants have experienced an accident of dental trauma. The most common rate of avulsion cases per month was zero cases followed by one to two cases. Higher rates of three to four or more than five cases per month showed lower rates (5.6% and 6.4%, respectively) (Table 2, Figure 4).

**Table 2:** Frequencies (n) and percentages (%) for responses to questions related to traumatic injury management (n = 392)

Traumatic injury management	n	%
Have you ever participated in educational courses focused on the management of traumatic tooth injuries?		
Yes	200	51
No	192	49
Have you ever experienced an accident of dental trauma?		
Yes	224	57.1
No	168	42.9
Number of cases of tooth avulsion per month:		
Zero	175	44.6
1-2 cases	170	43.4
3-4 cases	22	5.6
More than 5 cases	25	6.4

Knowledge Questionnaire

The highest number of correct answers was obtained with a question about splinting duration (79.8%), followed by a question about replacement of avulsed tooth in its socket (73.5%). The least number of correct answers was found with question regarding tooth management before replantation (13%), followed by question about duration of clinical and radiographic follow-up (15.3%).

The mean and standard deviation (SD) values for the knowledge score were 5.34 (1.65) with a minimum score of 1 and a maximum score of 9 (Table 3).

**Table 3:** Frequencies (n) and percentages (%) of correct responses and descriptive statistics for the total knowledge score (n = 392) Correct answers in bold. SD: standard deviation

Management of avulsion	Correct answers	
	n	%
Should an avulsed permanent tooth be replaced in its socket? (Not in all cases)	288	73.5
What is the best storage media? (Hank’s balanced salt solution)	223	56.9
What is the ideal extra-alveolar period? (<30 minutes)	243	62
What is the best systemic medication prescribed? (Prescribe antibiotics, anti-inflammatory drugs and tetanus prevention)	233	59.4
Tooth management before replantation (Hold the crown and wash with physiological saline solution)	51	13
Type of splint used is: (Flexible)	207	52.8
Splinting duration: (7 to 14 days)	313	79.8
Endodontic treatment of an avulsed tooth: (Depends on extra-alveolar period and stage of root formation)	260	66.3
Which one has better prognosis: (open apex or immature tooth)	216	55.1
Follow-up by clinical and radiographic examination for: (5 years)	60	15.3
Total score/10		
Mean (SD)	5.34 (1.65)	
Median (Range)	5 (1-9)	

Univariate analysis for knowledge scores

Association Between Demographic Data and Knowledge Scores

There was a statistically significant difference between the knowledge scores of males and females (P-value = 0.016). Females showed statistically significantly higher knowledge score than males. Moreover, there was a statistically significant difference between the knowledge scores of the Saudi and non-Saudi participants (P-value = 0.001). The Saudi participants showed a statistically significantly higher knowledge score than non-Saudis.

There was a statistically significant difference between the knowledge scores of public and private workers (P-value = 0.003). Dentists working in public hospitals showed a statistically significantly higher knowledge score than those working at private hospitals.

On the other hand, there was no statistically significant difference between the knowledge scores of different ages, qualifications, and experiences (P-value = 141, 0.178, 0.107, respectively (Table 4).

**Table 4:** Descriptive statistics and results of Mann-Whitney U test and Kruskal-Wallis test for the association between demographic data and knowledge scores * significant at P ≤ 0.05

Demographic data	Knowledge score					P-value
	Median	Minimum	Maximum	Mean	SD	
Gender						
Male	5	1	9	5.15	1.64	0.016*
Female	6	1	9	5.55	1.63	
Age						
24-30 y	5.5	1	9	5.37	1.66	0.141
31-40 y	5	2	9	5.38	1.59	
>40 y	4.5	2	7	4.42	1.51	
Qualifications						
Bachelor	5	1	9	5.35	1.64	0.178
Board	6	2	8	5.28	1.84	
Master	6	2	9	5.71	1.62	
PhD or Doctorate	5	2	8	4.74	1.51	
Nationality						
Saudi	6	2	9	5.5	1.6	0.001*
Non-Saudi	5	1	8	4.74	1.67	
Employment						
Public	6	1	9	5.57	1.69	0.003*
Private	5	1	8	5.05	1.54	
Experience						
Less than 5 years	5	1	9	5.36	1.64	0.107
6-15 years	5	1	9	5.45	1.67	
>16 years	4.5	2	7	4.43	1.5	

Association Between Education and Practice of Dental Traumatic Injuries and Knowledge Scores

The participants who have enrolled in an educational course regarding management of traumatic injuries to the teeth showed statistically significantly higher knowledge scores than those who have not attended (P-value = 0.043).

There was no statistically significant difference between the knowledge scores of participants who experienced or did not experience treatment of dental trauma cases (P-value = 386) (Table 5).

**Table 5:** Descriptive statistics and results of Mann-Whitney U test for the association between education and practice of dental traumatic injuries and knowledge scores * significant at P ≤ 0.05

Education and practice of dental traumatic injuries	Knowledge score					P-value
	Median	Minimum	Maximum	Mean	SD	
Have you ever participated in educational courses focused on the management of traumatic tooth injuries?						
Yes	6	1	9	5.47	1.73	0.043*
No	5	1	9	5.21	1.54	
Have you ever experienced an accident of dental trauma?						
Yes	5	1	9	5.42	1.64	0.386
No	5	1	9	5.24	1.66	

Multivariate analysis for knowledge scores

A linear regression model was constructed using knowledge scores as the dependent variable. Demographic data, attending traumatic injury management program, and experience of treating accidents of dental trauma were the independent variables. The results of the regression model showed that gender was a statistically significant predictor for knowledge scores (regression coefficient = -0.454, P-value = 0.007), with females showing higher knowledge scores than males. Nationality was a statistically significant predictor for knowledge scores (regression coefficient = -0.647, P-value = 0.003), with Saudis showing higher knowledge scores than non-Saudis. Employment was a statistically significant predictor for knowledge scores (regression coefficient = -0.458, P-value = 0.008), with those employed at public hospitals showing higher knowledge scores than those working at private hospitals. Finally, attending traumatic injury management programs was a statistically significant predictor for knowledge scores (regression coefficient = 0.376, P-value = 0.026), with participants who attended educational program showing higher knowledge scores than those who did not attend (Table 6).

**Table 6:** Results of the linear regression analysis model showing predictors of the knowledge scores * significant at P ≤ 0.05, SE: standard error

Variables	Unstandardized coefficients		P-value	95% confidence interval for B	
	B	SE		Lower Bound	Upper Bound
Gender	-0.454	0.166	0.007*	-0.780	-0.128
Age	-0.001	0.252	0.996	-0.496	0.494
Qualifications	0.019	0.102	0.851	-0.181	0.220
Nationality	-0.647	0.219	0.003*	-1.078	-0.217
Employment	-0.458	0.171	0.008*	-0.794	-0.122
Experience	-0.152	0.249	0.540	-0.642	0.337
Attending traumatic injury management programs	0.376	0.168	0.026*	0.046	0.706
Experienced dental traumatic injuries	0.212	0.169	0.212	-0.122	0.545

## Discussion

The present study involved a comprehensive analysis of 392 dentists, comprising 208 males (53.1%) and 184 females (46.9%). The majority of the participants, accounting for over three-quarters of the total, fell within the age bracket of 24-30 years. Approximately one-fifth of the participants were aged between 31 and 40 years, while a minor proportion, specifically 3.1%, were over 40 years old.

In terms of educational qualifications, the most prevalent degree among the participants was a bachelor's degree, encompassing more than three-quarters of the study cohort. Following this, master's degrees, PhDs, and board degrees exhibited progressively lower prevalence.

It is worth mentioning that a substantial majority of the participants, exceeding three-quarters, were Saudi nationals, highlighting the predominantly local representation in the study. Furthermore, approximately half of the participants were employed in public hospitals, highlighting the importance of their roles within the public healthcare system.

Regarding professional experience, the majority of the participants (83.9%) had less than five years of experience, indicating that a substantial portion of the sample consisted of relatively early-career dentists. A smaller fraction, 12.5%, reported 6-15 years of experience, while only 3.6% of the participants possessed experience exceeding 16 years. This distribution of experience levels within the sample can have implications for the interpretation of the study's findings, as it reflects varying degrees of clinical exposure and expertise among the participating dentists.

This study found that dentists that either finished their post-graduate studies or enrolled in a post-graduate program have significantly more knowledge toward managing and dealing with tooth avulsion compared to general dentists or dental students. This result is coinciding with comparable studies done in different demographic and geographical settings, which assert the importance of continuous education courses that focus on the management of dental emergencies [9,13,14]. Dental education levels and exposure to avulsion emergencies are particularly important as they are expected to be directly related to the knowledge and perception of tooth avulsion emergencies [10].

The analysis of the participants' responses to various questions revealed interesting insights into their knowledge levels regarding dental trauma management.

The question that received the highest number of correct answers was related to the duration of splinting, with a high correctness rate of 79.8%. This finding suggests that a substantial proportion of the surveyed dentists had a good understanding of the appropriate duration for splinting dental injuries. Following closely behind was the question concerning the replacement of an avulsed tooth in its socket, which garnered a correct response rate of 73.5%. This result indicates a commendable level of awareness among the participants regarding the immediate management of avulsed teeth.

However, it is worth noting that the study identified areas where knowledge appeared to be comparatively deficient. For instance, the question pertaining to tooth management before replantation yielded the lowest correct response rate at just 13%. This finding highlights a significant gap in understanding, potentially indicating the need for further education or training in this specific aspect of dental trauma management [10].

Similarly, the question regarding the duration of clinical and radiographic follow-up produced a correct response rate of 15.3%. This result suggests that there may be room for improvement in terms of dentists' knowledge about the long-term monitoring of patients who have experienced dental trauma.

Despite enormous efforts in structuring the questionnaire, the closed-end format of the items might have impacted the collected information. Future studies should consider including an open-end option to allow participants to share their experience.

## Conclusions

Higher dental education and post-graduate training gave the dentists better judgment of dealing with dental emergencies compared to general dentists. Thus, the recommendation of this study is to include the comprehensive treatment of avulsion emergencies in the dental school curriculums, increase the exposure of dental students to such cases, and add simulation cases and other interactive courses into the curriculum that represent the real emergency scenario.
